# A Case of Complete Remission from Advanced Gastric Adenocarcinoma with Synchronous Liver Metastasis in Response to EOX Chemotherapy

**DOI:** 10.1155/2018/3954260

**Published:** 2018-10-23

**Authors:** Zhi-jun Yan, Jia-xin Wang, You-yi Liu, Zhen-hui Li, Li-juan Zhang, Ping Gan, Xiao Han, Li-kun Luan, You-guo Dai

**Affiliations:** ^1^Department of Abdominal Surgery, Third Affiliated Hospital of Kunming Medical University, Kunming, Yunnan 650118, China; ^2^Department of Radiology, Third Affiliated Hospital of Kunming Medical University, Kunming, Yunnan 650118, China; ^3^Department of Pathology, Third Affiliated Hospital of Kunming Medical University, Kunming, Yunnan 650118, China

## Abstract

Gastric cancer is a malignant tumor with a high degree of malignancy. Multiple liver metastases from gastric cancer (LMGCs) are common. However, the treatment of LMGCs is very difficult. It is rare to achieve complete remission (CR) and long-term survival after treatment. We present the case of a patient with gastric adenocarcinoma and multiple liver metastases who showed CR for more than 33 months after perioperative EOX (epirubicin, oxaliplatin, and capecitabine) combination chemotherapy with radical distal gastrectomy and resection of liver metastases. The patient is still in follow-up without tumor recurrence. These findings suggest that LMGC does not necessarily mean a poor prognosis; preoperative chemotherapy combined with surgery may be a good treatment option for LMGC in selected patients. Further studies are needed to support this treatment approach.

## 1. Introduction

Gastric cancer is a common gastrointestinal malignancy originating from the epithelium and considered the fifth most commonly occurring cancer and the second leading cause of cancer-related deaths worldwide [1]. Gastric cancer incidence rates have been declining in most parts of the world in recent decades. However, in 2012, gastric cancer caused more than 320,000 deaths in China, accounting for approximately 45% of stomach cancer-related deaths worldwide, representing a heavy disease burden of stomach cancer in the country [2]. The main reason is that 80% of gastric cancer is in advanced stage when diagnosed; thus, the 5-year survival rate is low. According to statistics from 2010 to 2014, the 5-year survival rate of gastric cancer in China is only 35.9%. In contrast, survival was remarkably high in South Korea (68.9%) and Japan (60.3%) during the same period [3].

Liver metastases from gastric cancer (LMGCs) predict poor prognosis and are present in 40% of patients dying from this disease [4]. There are few surgical opportunities for patients with LMGCs because of polycentric or extraliver disease [5]. However, data from retrospective studies with a relatively small number of subjects suggest that surgical resection for some patients with LMGCs is feasible and beneficial to long-term survival [6]. With the development of chemotherapeutic research for gastric cancer, it has been reported that some patients with LMGC received R0 resection with long-term survival after preoperative chemotherapy [7]. Unfortunately, only rare reports have presented good therapeutic results for LMGC treated with chemotherapy and/or surgical treatment.

The objective of this case report is to explore the role of preoperative chemotherapy combined with surgical resection in the selected patients of LMGC. Here, we report a case of LMGC that showed CR for more than 33 months after having preoperative treatment with EOX (epirubicin, oxaliplatin, and capecitabine) combination chemotherapy followed by radical distal gastrectomy and resection of metastases to the liver.

## 2. Case Report

A 52-year-old man presented with upper abdominal pain for more than 3 months and weight loss of 3 kg in 10 days in July 2015. His performance status was 1 according to the criteria of the Eastern Cooperative Oncology Group (ECOG). The blood count analysis results were as follows: white blood cell (WBC) 23.99 × 10^∧^9/L, neutrophil (NEUT) 86.1%, and absolute neutrophil count (ANC) 20.64 × 10^∧^9/L. The tumor marker test results were as follows: serum carcinoembryonic antigen (CEA) 177 ng/dl, carbohydrate antigen-724 (CA-724) 20.34 IU/ml, and carbohydrate antigen-153 (CA-153) 31.59 IU/ml. Bone marrow biopsy of the granulocyte series demonstrated obvious active hyperplasia, and megakaryocytic and erythrocytic series were active and proliferous. There was no evidence of bone marrow metastasis.

**Figure 1:**
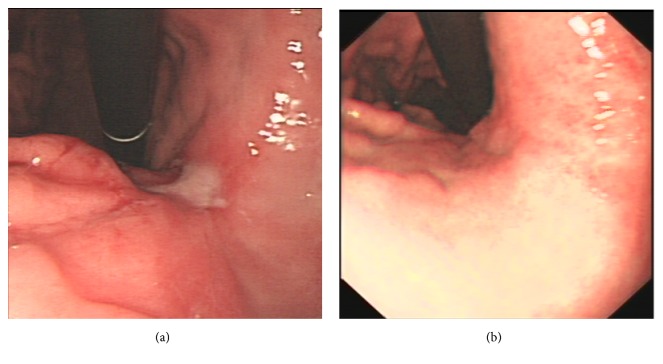
**Initial (a) and follow-up (b) endoscopic findings after six cycles of EOX chemotherapy**. (a) A large ulceroinfiltrative mass at the anterior wall of the lesser antral curvature, covered with whitish fur, and no apparent active bleeding; (b) ulcer lesion disappeared and replaced by scar.

**Figure 2:**
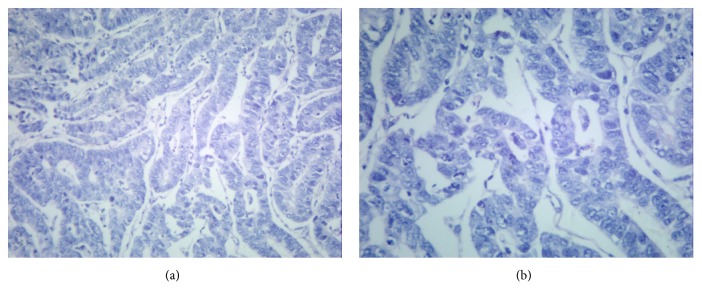
**Microscopic findings at the time of diagnosis. Well differentiated tubular adenocarcinoma** ((a) H&E, ×100; (b) H&E, ×200).

**Figure 3:**
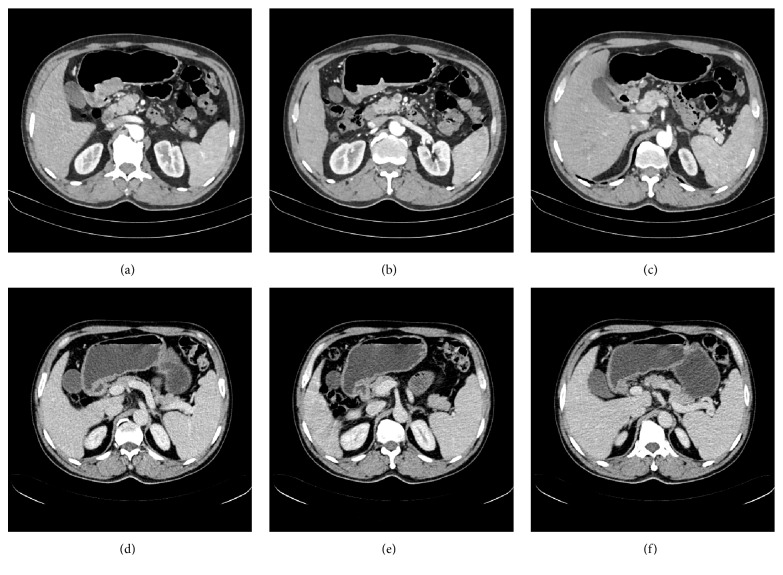
**Initial abdominal computed tomography scan (a~c) and follow-up scan after three cycles of EOX chemotherapy (d~f)**. (a, b, and c) Irregular wall thickening on the lesser curvature side of the gastric antrum with mass formation, large ulceroinfiltrative, and multiple metastases to neighboring lymph nodes; (d, e) scan shows that the mass of the gastric antrum decreased in range from approximately 7 cm to less than 3.3 cm; (f) disappearance of metastatic lesion of neighboring lymph nodes that had been previously observed.

**Figure 4:**
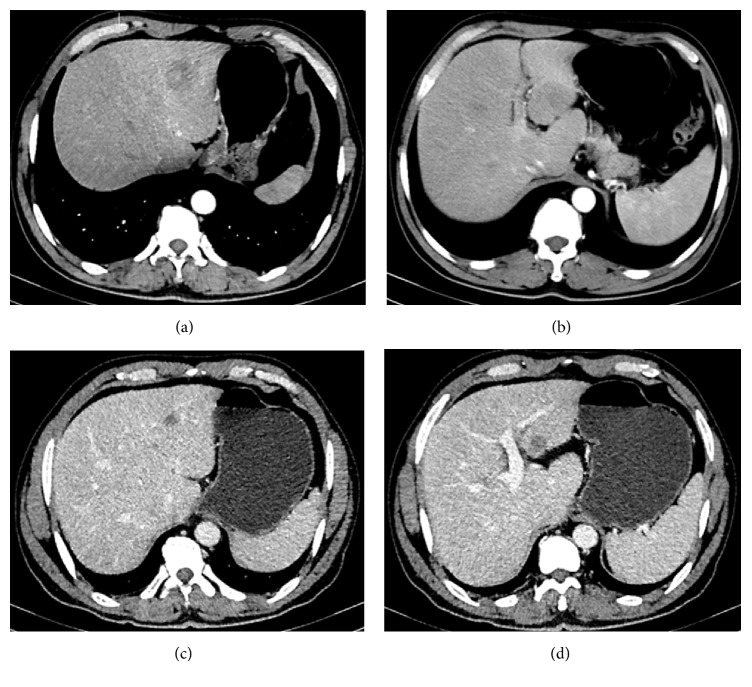
**Initial abdominal computed tomography scan (a, b) and follow-up scan after three cycles of EOX chemotherapy (c, d)**. (a) Scan shows that the metastatic lesion in liver segment 4; (b) hypodense metastatic lesions in the left lobe of liver; (c) scan shows that the metastatic lesion in liver segment 4 has decreased in size from 2.8 cm to less than 0.9 cm; (d) scan shows that the metastatic lesion in the left lobe of liver has decreased in size from 4.3 cm to less than 1.5 cm.

Upper gastrointestinal endoscopic findings showed 3 cm × 2 cm ulcers at the anterior wall of the lesser antral curvature with no apparent active bleeding (Figures 1(a) and 1(b)). Histopathological examination revealed a well differentiated tubular adenocarcinoma (Figure 2). An abdominal computed tomography (CT) scan demonstrated irregular wall thickening on the lesser curvature side of the gastric antrum with mass formation with a large ulceroinfiltration as well as multiple metastases to neighboring lymph nodes (Figures 3(a)–3(c)). The CT scan also revealed metastatic lesions in liver segment 4 (Figure 4(a)) and evidence of hypodense liver metastatic lesions in the left lobe of the liver (Figure 4(b)). The CT scan also showed multiple liver metastases lesions that ranged in size from 1.0 to 4.3 cm over the entire liver (Figures 4(a) and 4(b)).

A clinical diagnosis of stage IV (cT3NxM1) advanced gastric cancer was made according to the 7th American Joint Committee on Cancer (AJCC) system. Trastuzumab plus cisplatin-based chemotherapy has been recommended as the first-line standard treatment regimen for the patients with HER2-positive advanced gastric cancer according to the 2015.V3 gastric cancer guidelines of the National Comprehensive Cancer Network (NCCN). Since the result of HER2 status testing in this case was negative, it was not necessary to use the drugs targeting HER2, such as trastuzumab, for this patient.

On the basis of the abovementioned findings, we administered EOX combination chemotherapy. For each cycle, intravenous infusion epirubicin (50 mg/m^2^) was administered on day 1, followed by an intravenous drip of oxaliplatin (130 mg/m^2^) for 2 hours on day 1. Oral capecitabine (625 mg/m^2^) was administered twice daily for 3 weeks. This regimen was repeated every 3 weeks.

**Figure 5:**
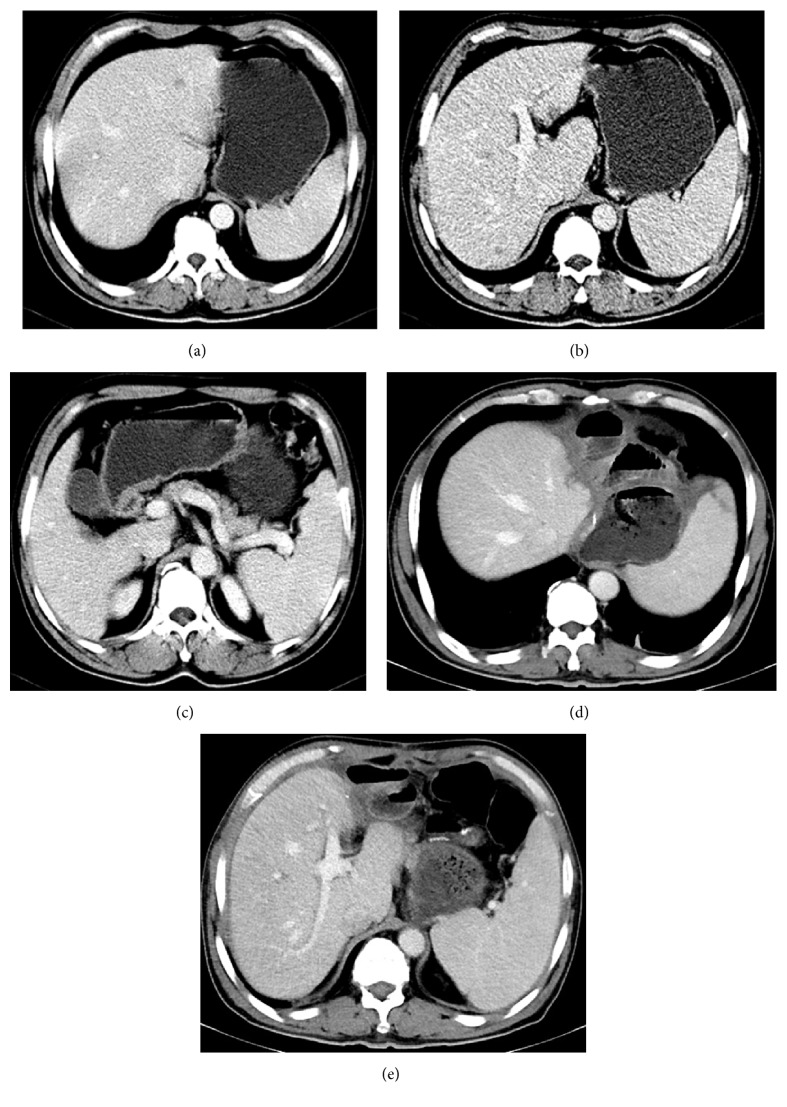
**Follow-up abdominal computed tomography scans after six of EOX chemotherapy (a, b, c) and 27 months after surgery (d, e)**. (a) The liver metastatic nodules in liver segment 4 that had been previously observed have almost disappeared; (b) the liver metastatic lesion in the left lobe of liver that had been previously observed has evidently been reduced; (c) the gastric mass has almost disappeared; (d) postradical distal gastrectomy with Roux-en-Y with residual stomach and jejunum anastomosis and D2 lymphadenectomy combined with liver metastatic lesions resection, with no evidence of tumor recurrence 27 months after surgery; (e) no recurrence of metastatic lesions after 27 months.

In October 2015, after completion of three cycles of chemotherapy, an abdominal CT scan showed that the mass of the gastric antrum had decreased to less than 3.3 cm (Figures 3(d) and 3(e)), and metastatic lesions of neighboring lymph nodes that had been previously observed had disappeared (Figure 3(f)). The scan showed that the liver metastatic lesions had almost disappeared, with the exception of lesions in the left lobe of the liver, which measured less than 1.5 cm in size (Figures 4(c) and 4(d)). After an additional three cycles of chemotherapy, another abdominal CT scan was performed in December 2015. In December 2015, after completion of six cycles of chemotherapy, endoscopic findings showed improvement in the gastric antrum mass. This result showed that the liver metastatic nodules that had been previously observed were no longer present (Figures 5(a) and 5(b)). The scan showed that the gastric mass had almost disappeared (Figure 5(c)). Follow-up endoscopy showed that the ulcer lesion had disappeared and was replaced by scar tissue (Figure 1(b)).

Radical distal gastrectomy with Roux-en-Y with a residual stomach and jejunum anastomosis, as well as a D2 lymphadenectomy, combined with obvious metastatic liver lesions resection was performed in January 2016, resulting in complete removal of the primary tumor and any suspicious lymph nodes. Pathological findings after surgery showed no tumor cells detected in the gastric primary lesion. Metastases to perigastric lymph nodes were observed in none of 18 resected lymph nodes, suggesting pathological complete remission. The final pathological stage was ypT0N0M0. Following the operation, we planned to administer postoperative adjuvant chemotherapy with another two cycles of EOX regimen again.

The side effects and toxicities were evaluated every regimen cycle. During EOX treatment, the patient presented some adverse events such as mild gastrointestinal reaction, grade 1 neuritis, and grades 1-2 hematological toxicities that were considered tolerable. Mild gastrointestinal reactions, including grade 1-2 nausea and vomiting, were the most common EOX-related toxic effects.

Every 3 months, an abdominal CT scan was performed. There was no evidence of recurrent tumor up to February 2018. The patient had maintained CR for more than 27 months after surgery, with a 33-month overall survival (Figures 5(d) and 5(e)). The patient still had no tumor recurrence up to the time of this case history article submission.

## 3. Discussion

The liver is the most common site for metastases in patients with gastric cancer. It has been reported that approximately 14% of gastric cancer patients have developed synchronous liver metastasis and 25% to 30% of gastric cancer patients have developed metachronous liver metastasis after surgical resection of the primary gastric cancer [8, 9]. There were no effective treatments for patients of LMGCs, and the prognosis of these patients remains very poor [10]. The existing literature suggests that the 5-year survival of gastric cancer patients with liver metastasis is less than 10% [11] and that the median survival rate of patients treated with gastrectomy alone has been reported to range from 3.4 to 5.7 months [10]. There is no significant difference in survival between synchronous and metachronous metastasis [12].

The value of liver resection for LMGC remains controversial. Obviously, the number of liver metastases is a considerable prognostic factor for survival after surgery, and patients with a solitary metastasis of gastric cancer are good candidates for surgical resection [13]. Unfortunately, most LMGCs are multiple, bilateral, and combined with peritoneal or lymph node metastases that directly invade adjacent organs; as a result, hepatectomy was found to be indicated in only 0.4-1.0% of patients with LMGCs [14]. Therefore, surgical resection requires good indications; otherwise, it is difficult to achieve satisfactory results for LMGCs.

Recently, in addition to postoperative chemotherapy [15], preoperative chemotherapy has also been used as a standard treatment for advanced gastric cancer [16]. However, since it is very difficult to achieve CR in metastatic gastric cancer, palliative chemotherapy is the main treatment option [17]. In a review of the literature, preoperative chemotherapy has been recommended as the preferred treatment pathway for advanced gastric cancer because of the advantages of tumor downstaging, increased tumor resection rate, and improved survival rate [18–20]. The MAGIC study of resectable gastroesophageal cancer showed that ECF (epirubicin, cisplatin, and fluorouracil/leucovorin) perioperative chemotherapy significantly improved progression free survival (PFS) and OS compared with surgery alone [18]. Furthermore, the REAL-2 study found that perioperative EOX chemotherapy was noninferior to perioperative epirubicin, cisplatin, and 5-FU/CF (ECF) chemotherapy in efficacy and median survival of patients suffering gastroesophageal cancer [21]. Inspired by these results, we used EOX in preoperative chemotherapy for the patients of LMGC who had a better performance status.

We have presented a case of a patient with advanced gastric adenocarcinoma with synchronous and multiple liver metastases who was treated with preoperative EOX combination chemotherapy followed by radical distal gastrectomy and resection of metastatic liver lesions resulting in a CR of more than 33 months.. This case suggests that preoperative EOX chemotherapy combined with surgery may be a good treatment option for LMGC. However, to achieve better results, patients with LMGC should have a good ECOG score, and the number of liver metastases should preferably be less than 3. Indeed, there are many problems that need to be further explored after preoperative chemotherapy for LMGC. For example, there is still lack of evidence as to whether patients with CR need to continue chemotherapy after surgery. According to the current consensus of some experts [22], it is recommended to continue the original chemotherapy program after surgery. However, it is difficult for most patients to tolerate the 3-drug chemotherapy because of a relatively poor performance status after surgery. Therefore, dosage adjustment or reduction in drug administration should be considered after surgery. In addition, liver metastases reduced or disappeared after preoperative chemotherapy. At this time, how to determine the extent of hepatectomy and how to determine the extent of the original lesions are also urgent problems to be solved. Recently, a study of the German AIO study group investigating a perioperative FLOT regimen (fluorouracil, leucovorin, oxaliplatin, and docetaxel) versus ECF/X demonstrated higher rates of pathological response for FLOT (15.6% versus 5.8%) and lower rates of one serious adverse event involving a perioperative medical or surgical complication (25% versus 40%) [23]. Based on these results, in the future, we will also consider the use of FLOT chemotherapy in the preoperative chemotherapy of LMGCs.

## 4. Conclusion

This case suggests that preoperative chemotherapy combined with surgery may be a good treatment option for LMGC in selected patients. However, further clinical trials are required to evaluate the survival benefit, efficacy, and side effects of this therapeutic regimen.
